# Is nasal septal suturing an alternative technique to nasal packing? A protocol for systematic review and meta-analysis

**DOI:** 10.1097/MD.0000000000023535

**Published:** 2020-12-11

**Authors:** Dandan Wang, Ting Liu, Chao Liao, Guangjun Tang, Tian Tian, Li Tian

**Affiliations:** a Hospital of Chengdu University of Traditional Chinese Medicine; b Chengdu University of Traditional Chinese Medicine, Chengdu, Sichuan Province, PR China.

**Keywords:** clinical trial, meta-analysis, nasal packing, nasal septal suturing, protocol, septoplasty, systematic review

## Abstract

**Background:**

Nasal septal suturing is a commonly used alternative treatment to nasal packing after septoplasty. Besides alleviating postoperative discomfort, extensive studies have shown that nasal septal suturing is more effective than nasal packing. However, its clinical benefits remain controversial.

**Methods:**

We will perform a systematic review of nasal packings effect-related outcome in comparison to nasal septum suture among septoplasty patients by searching 8 databases, based on the Preferred Reporting Items for Systematic Reviews and Meta-Analyses (PRISMA). All eligible studies will be screened against the inclusion and exclusion criteria. Two independent reviewers will extract the data. Moreover, Review Manage 5.3 will be used for quality assessment and data analysis. Then, the random effects model or fixed effects model will be applied according to the heterogeneity. In conformity with the GRADE criteria, the merits of the evidence and recommended strength will be assessed.

**Results:**

This protocol will guide subsequent systematic reviews and meta-analyses. The differences in efficacy between nasal septal suturing and nasal packing after septoplasty will be evaluated in terms of efficiency, adverse reaction, comfort degree, and other factors.

**Conclusion:**

This proposed study will explore the possibility of adopting nasal septal suturing as an alternative to nasal packing after septoplasty.

**OSF registration number:**

doi: 10.17605/OSF.IO/WF3GX.

## 1 Introduction

Given that nasal septum bends to 1 or 2 sides and protrudes locally, nasal septum deviation is a prevalent disease in otorhinolaryngology. Consequently, it causes symptoms such as nasal obstruction, epistaxis, headache, as well as dysfunctions in the nasal cavity, paranasal sinus, and middle ear.^[1,2]^ Septoplasty, the most common otorhinolaryngology procedure in adults,^[3]^ is currently the only recognized treatment for septal deviation. With the extensive development of nasal endoscopic surgery, it has become increasingly common and advanced both locally and internationally, resolving problems like stuffy nose and headache.^[4]^ The postoperative nasal septum was often fixed with the Vaseline gauze or expanded sponge to prevent postoperative hemorrhage and the adhesion of nasal septum, stabilize the internal structure of the nasal cavity and prevent recurrence.^[5]^ However, mechanical pressure generated by packing can destroy mucociliary activity, block lymphatic vessels and cause lymphedema.^[6]^ This foreign body in the nose can cause stuffiness and discomfort, and even worse, induce rhinocardiac reflex.^[7]^ Upon the packings removal, the patient evidently suffers from pain or bleeding again, which far exceeds the pain caused by the trauma of the surgery itself. Equally important, the patients breathing, quality of sleep and emotional state are all affected. Although nasal packing is frequently utilized in nasal surgery, its use as a routine measure has been questioned.^[8]^

Alternatively, nasal septal suturing can avoid the pain caused by packing and removal, repair the torn mucosa, fix the nasal septum cartilage, and alleviate mucosal edema. It is a treatment that is regularly performed after septoplasty.^[9,10]^ However, some studies have demonstrated that nasal septal suturing has no superiority over nasal packing in preventing postoperative adhesion, edema and infection.^[11,12]^ Various meta-analyses examined the efficacy and advantages of the former over the latter in preventing complications such as adhesion, hematoma, bleeding, perforation, infection, and residual deflection, as well as in making patients more comfortable by eliminating or minimizing postoperative headache and pain.^[13,14]^ Nevertheless, given their small sample sizes and incomplete outcome indicators, these meta-analyses are inadequate, requiring large-sample multi-center randomized controlled trials to provide higher-quality evidence. In recent years, multiple clinical studies of nasal septal suturing versus nasal packing in China and abroad have taken place, highlighting the prevailing controversy. Thus, the extensive evidence of their efficacy, comfort, and complications must be explored to provide a more cohesive and holistic meta-analysis, which is fundamental in clinical decision making.

## 2 Methods/design

This study had been registered at Open Science Framework (OSF) (https://osf.io/wf3gx). The registration number is DOI 10.17605/OSF.IO/WF3GX.

### 2.1 Search strategy

We performed a literature search using PubMed, Embase (Excerpta Medical Database), the Cochrane Library, the Chinese Cochrane Centre's Controlled Trials Register platform, the Wanfang Chinese Digital Periodical and Conference Database, the China National Knowledge Infrastructure (CNKI) database, and the VIP Chinese Science and Technique Journals Database through February 29, 2020.

The additional sources will include the Chinese Clinical Trial Registry (ChiCTR) and the references of the included studies. The eligible studies will be selected according to the inclusion criteria. If the data were incomplete, we would communicate with the authors of the studies to acquire the required and essential information. Table 1 itemizes the exhaustive search strategy for PubMed. Comparable search strategies will be established for different databases.

**Table 1:** Search strategy for PubMed.

Number	Search terms
#1	pack^∗^
#2	trans^∗^ AND sutur^∗^
#3	septal suture
#4	intranasal septal splints
#5	#2-#4/OR
#6	septoplasty
#7	#1 AND #5 AND #6

### 2.2 Inclusion and exclusion criteria

#### 2.2.1 Inclusion criteria

1. Studies: Only prospective randomized controlled trials (RCTs) of nasal septal suturing versus nasal packing after septoplasty will be selected. Eligible languages were limited to Chinese or English. The inclusion criteria will be broadened when fewer than 5 qualified RCTs are found for this systematic review.
2. Participants: All patients diagnosed with septal deviation and suffering from septoplasty will be included.
3. Interventions: The postoperative intervention in the experimental group will be nasal septal suturing. The control method will be nasal packing, which involves a variety of materials like Vaseline gauze, self-expanding polyvinyl acetate packs, silicone plate, fibrin glue, and absorbable gelatin^15^.
4. Outcomes: The pre-specified primary outcomes will be the clinical effectiveness and visual analogue score (VAS) for comfort (e.g., nasal congestion, headache, difficulty swallowing, and sleep disorders). Adverse events such as adhesion and septal hematoma will be incorporated in the secondary outcomes. During the review, these pre-specified outcomes will be modified based on the findings of this papers references. If the results are used in our systematic review, special attention will be undertaken to avoid selective reporting bias.

#### 2.2.2 Exclusion criteria

The exclusion criteria are

1. patients with partial turbinectomy and functional endoscopic sinus surgery,
2. incomplete data that remained inaccessible after reaching out to the authors of original studies, and
3. duplicate data and repeatedly published studies.

### 2.3 Data abstraction

**Figure 1:**
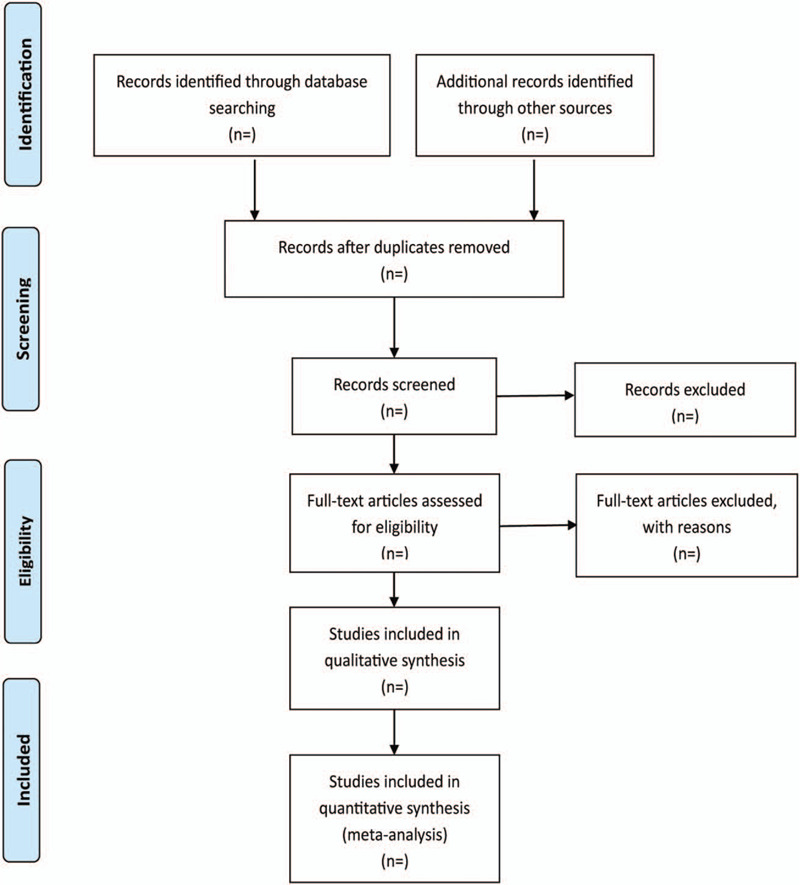
Flowchart of study selection.

All articles obtained by the above search strategy were imported into Endnotes X9 to eliminate duplicate studies. In compliance with the inclusion and exclusion criteria, 2 independent investigators will screen the abstracts of the articles after performing a retrospective analysis on the fully screened texts. The finalized set of selected articles will be organized in Microsoft Excel. Additionally, 2 authors will independently extract raw data from the qualified articles, including study region, publication and follow-up information, author details, sample size, outcome measures, and intervention and control methods. Then, a third investigator will validate the extracted data to ascertain the completeness and accuracy. The outcome variables will be collected for all included studies. Conflicts that arise will be settled through discussions or arbitrations by third-party reviewers. Figure 1 illustrates the Preferred Reporting Items for Systematic Reviews and Meta-Analyses (PRISMA) that we developed in line with the eligibility criteria and search strategy. It also provides this papers study flow diagram.

### 2.4 Quality assessment

Based on the Cochrane Handbook for Systematic Reviews of Interventions, 3 investigators will use the Review Manager (RevMan) software version 5.3 to evaluate the included RCTs, specifically their methodological quality. Moreover, this assessment will be performed according to 7 criteria: allocation concealment, selective outcome reporting (SOR), other risks of bias, incomplete outcome data, randomization generation, as well as blinding of study personnel/patients and outcome assessors.^[16]^ Among the different intervention groups with distinct baseline characteristics, trials sponsored by the packaging manufacturer will be defined as “other bias”. If at least 10 trials report the primary outcomes, we will evaluate publication bias using funnel plots.

### 2.5 Statistical analysis

For the meta-analysis, RevMan 5.3 will be utilized to summarize our data. Along with 95% confidence intervals (CIs), the relative risks will depict the measurements of dichotomous variables. Moreover, the statistical significance will be set at *P* < .05, and continuous data will be demonstrated as the mean difference together with 95% CIs. In this systematic review, interstudy heterogeneity will be assessed through the *I*^2^, where *P* ≤ .1 and *I*^2^ > 50% represent considerable heterogeneity. We will also apply the random effects model and subgroup analysis.

When the data heterogeneity is low at *P* ≥ .1 and *I*^2^ < 50%, we will use the fixed effects model (FEM) to merge. Then, when the packing materials of such studies are similar, the trials will be summarized. Based on these similarities, specific subgroups will be examined. A sensitivity analysis will be executed if the *P* value test for heterogeneity after the subgroup analysis is less than 1 to evaluate the robustness of the results. After eliminating the substandard studies, the meta-analysis will be performed again. Additionally, we will investigate the impact of statistical models on the results. The statistical significance will be established at *P* < .05.

### 2.6 Grading of recommendations assessment, development and evaluation quality assessment

In conformity with the Grading of Recommendation Assessment, Development and Evaluation (GRADE) criteria, 2 independent reviewers will assess the confidence levels of the outcomes. In most instances, differences of opinion will be settled through discussions. Otherwise, a consultation with a third reviewer will take place prior to making a final decision.

## 3 Discussion

Some RCTs have shown that nasal packing after septoplasty can manage the corrected nasal septum, compress and stop the bleeding, and prevent hematoma.^[17]^ However, whether using a Vaseline gauze or high-expansion hemostatic sponge packing, it has caused many discomforts in patients, such as unforgettable nasal pain and headache.^[18,19]^ However, studies have confirmed that nasal packing after septoplasty has no significant advantage in reducing postoperative bleeding, perforation of the nasal septum, and adhesion.^[15]^ Moreover, it has been proposed that it is not an essential treatment after septoplasty, and its clinical value has been disputed.

Alternatively, nasal septal suturing can effectively reduce the shortness of breath caused by nasal packing and the severe pain and anxiety caused by its removal.^[20]^ However, studies have found that this treatment increases the risk of bleeding and hematoma.^[21]^ In this case, nasal septal suturings superiority over nasal packing after septoplasty remains controversial. While previous meta-analyses have shown that nasal septal suturing is a first-line treatment alternative to conventional nasal packing,^[13]^ more well-designed studies are still needed to establish its clinical efficacy. In recent years, ongoing randomized controlled trials have generated new evidence for nasal septal suturings clinical application.^[11,22,23]^ Therefore, there is an urgent need to further update and supplement the systematic review by including more research results in the analysis to improve the evidence for nasal septal suturing in the treatment after septoplasty. Furthermore, we estimate that this systematic review may have 4 potential limitations:

1. The indicators of some studies are not comprehensive.
2. Several important indicators can cause heterogeneity due to different measurement methods.
3. There is no systematic or quantitative description of the baseline indicators.
4. There is no consensus on the follow-up time, and the quality of the included studies is not up to standard.

As our project focuses on these issues, we will use descriptive methods to present our findings where necessary. This systematic review will be based on study data or results in the existing published and unpublished articles that have observed differences between the efficacy of nasal septal suturing and nasal packing after septoplasty. Before proceeding with the study, we hope that the dissemination of this protocol will provide us with constructive feedback and suggestions regarding the most suitable methods.

Ultimately, the proposed systematic review and meta-analysis will supply evidence-based support for the efficacy and advantages of nasal septal suturing over nasal packing after septoplasty. The results are likely to substantiate the national and international guidelines for the treatment of patients with septoplasty. The review will also highlight the need for further research on this topic.

## Author contributions

**Conceptualization:** Dandan Wang, Guangjun Tang.

**Data curation:** Dandan Wang, Ting Liu.

**Formal analysis:** Dandan Wang, Ting Liu.

**Investigation:** Ting Liu, Chao Liao, Dandan Wang.

**Methodology:** Dandan Wang, Ting Liu.

**Resources:** Li Tian.

**Software:** Chao Liao, Tian Tian.

**Supervision:** Li Tian, Guangjun Tang, Tian Tian.

**Validation:** Dandan Wang, Tian Tian.

**Writing – original draft:** Dandan Wang.

**Writing – review & editing:** Dandan Wang, Li Tian.
